# Reinforcer value moderates the effects of prenatal alcohol exposure on learning and reversal

**DOI:** 10.3389/fnins.2023.1147536

**Published:** 2023-04-25

**Authors:** Jayapriya Chandrasekaran, Belkis Jacquez, Jennifer Wilson, Jonathan L. Brigman

**Affiliations:** ^1^Department of Neurosciences, University of New Mexico School of Medicine, Albuquerque, NM, United States; ^2^New Mexico Alcohol Research Center, UNM Health Sciences Center, Albuquerque, NM, United States

**Keywords:** reward value, touchscreen, development, FASD, non-nutrient sweetener

## Abstract

**Introduction:**

Fetal Alcohol Spectrum Disorders (FASD) are the leading cause of preventable developmental disability and are commonly characterized by alterations in executive function. Reversal learning tasks are reliable, cross-species methods for testing a frequently impaired aspect of executive control, behavioral flexibility. Pre-clinical studies commonly require the use of reinforcers to motivate animals to learn and perform the task. While there are several reinforcers available, the most commonly employed are solid (food pellets) and liquid (sweetened milk) rewards. Previous studies have examined the effects of different solid rewards or liquid dietary content on learning in instrumental responding and found that rodents on liquid reward with higher caloric content performed better with increased response and task acquisition rate. The influence of reinforcer type on reversal learning and how this interacts with developmental insults such as prenatal alcohol exposure (PAE) has not been explored.

**Methods:**

We tested whether reinforcer type during learning or reversal would impact an established deficit in PAE mice.

**Results:**

We found that all male and female mice on liquid reward, regardless of prenatal exposure were better motivated to learn task behaviors during pre-training. Consistent with previous findings, both male and female PAE mice and Saccharine control mice were able to learn the initial stimulus reward associations irrespective of the reinforcer type. During the initial reversal phase, male PAE mice that received pellet rewards exhibited maladaptive perseverative responding whereas male mice that received liquid rewards performed comparable to their control counterparts. Female PAE mice that received either reinforcer types did not exhibit any deficits on behavioral flexibility. Female saccharine control mice that received liquid, but not pellet, rewards showed increased perseverative responding during the early reversal phase.

**Discussion:**

These data suggest that reinforcer type can have a major impact on motivation, and therefore performance, during reversal learning. Highly motivating rewards may mask behavioral deficits seen with more moderately sought rewards and gestational exposure to the non-caloric sweetener, saccharine, can impact behavior motivated by those reinforcers in a sex-dependent manner.

## Introduction

Fetal alcohol spectrum disorders (FASDs) are the leading cause of preventable developmental disability (Popova et al., 2017, 2018) and are characterized by deficits in executive function (Chudasama, 2011). Findings in children with FASD suggest that difficulties in planning, cognitive flexibility, and inhibition are better predictors of behavioral problems than intelligence-based measures (Mattson et al., 1999; Kodituwakku et al., 2001). Behavioral flexibility, one of the core dimensions of executive functioning, is crucial for an individual to adapt to the ever-changing environment. Impairment in behavioral flexibility is a common feature across neuropsychiatric and neurodevelopmental disorders, including FASD, and has also been documented in prenatal alcohol exposure (PAE) rodent models (Elliott, 2003; Holmes and Wellman, 2009; Chudasama, 2011). Reversal learning paradigms, which are widely used for assessing behavioral flexibility across species, have been shown to be consistently impaired by PAE across numerous routes of alcohol administration, doses, and assessment modalities including spatial, operant, tactile, and olfactory (Wainwright et al., 1990; Thomas et al., 2004; Marquardt et al., 2014; Atalar et al., 2016; Marquardt and Brigman, 2016; Waddell and Mooney, 2017).

Over the past ~20 years, numerous studies have demonstrated that the formation of a stimulus-outcome association during discrimination learning is mediated by the dorsal striatum (DS), whereas the reversal of these associations is mediated by cortical areas including the orbital frontal cortex (OFC) (Brigman et al., 2010c; Graybeal et al., 2011). For example, *in vivo* electrophysiology studies have shown that the DS mediates associative learning *via* the integration of state-action-outcome associations (Yin et al., 2009; Corbit et al., 2012; Brigman et al., 2013; Bergstrom et al., 2018), while the OFC encodes response outcomes and tracks changes in choice value (Schoenbaum et al., 2003; Bissonette et al., 2008; Moorman and Aston-Jones, 2014; Marquardt et al., 2017). Regardless of modality (e.g., spatial or operant), a common feature of most reversal tasks is the need to reinforce choice decisions (Bussey et al., 2008; Horner et al., 2013), while there are several reinforcers available, most operant tasks employ reinforcers either solid (e.g., dustless pellets) or increasingly, liquid sweetened reward (e.g., strawberry milkshake).

It has been increasingly recognized that reward type can have a major influence on incentive motivation to both learn an initial association and reverse previously learned associations. There is a broad range of reinforcers available from solid to liquid reinforcers (Horner et al., 2013), and several reports confirm that both the type and amount of the reinforcer affect the performance of rodents on various tasks used to assess cognition (Skjoldager et al., 1993; Eagle et al., 1999; Youn et al., 2012; Hutsell and Newland, 2013). The ability of three different inbred mice strains (BALB/c, C57BL/6, DBA/2) to respond on a fixed reinforcement schedule using qualitatively different reinforcers such as flavored pellets or milk was examined. Interestingly, mice reinforced with milk had higher response rates when compared to mice reinforced with pellets on moderate ratios (Hutsell and Newland, 2013).

Strawberry milkshake has been more commonly used as a reinforcer in touchscreen-based tasks (Phillips et al., 2017) following informal observations about it being a powerful reinforcer when testing cognition and behavior (Horner et al., 2013; Mar et al., 2013). Using touchscreen-based pairwise discrimination and reversal tasks, the reinforcer strength of strawberry milk was compared to that of super saccharine (1.5% or 2% (w/v) saccharin solution). Mice on strawberry milk acquired the task faster and performed fewer errors when compared to the mice on super saccharine, thus reaffirming the superior strength of strawberry milk and confirming the differential role of the type of reinforcement of the performance of behavioral assessment (Phillips et al., 2017). Furthermore, there exists diversity in the type of strawberry milk used based on its caloric content and the sweeteners it contains (Kim et al., 2017). It was observed that the caloric content of the liquid reinforcer determined the incentive value rather than the fat content, sugar content, or flavor of the reinforcer (Kim et al., 2017). Youn et al. (2012) reported a difference in performance on spatial memory tasks between two genetically different mouse strains and determined that it was due to differential reinforcer impact rather than genetic differences.

Previously, we have examined the effects of PAE on behavioral flexibility utilizing a touchscreen reversal task motivated *via* flavorless dustless 14 mg pellets. We showed that male and female PAE mice exhibit behavioral inflexibility, as measured by increased perseverative responses during the early stages of a reversal task (Marquardt et al., 2014, 2020). Given the increasing awareness that reinforcer type can alter performance on various behavioral tasks, we examined whether a more motivating reinforcer, such as strawberry milk, would change the performance of the PAE mice on the touchscreen-based discrimination and reversal task.

## Materials and methods

### Prenatal alcohol exposure model

**Figure 1:**
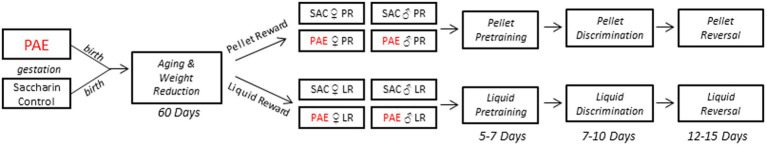
Experimental timeline of exposure and operant testing. Following weaning, mice were allowed to age to 8 weeks prior to weight reduction and assignment to experimental reward groups. Mice then underwent pretraining to initiate and respond to visual stimuli. Following the acquisition of task behaviors, all mice underwent testing on pairwise discrimination followed by reversal learning. LR, liquid reward; PR, pellet reward.

Male and female C57BL/6J mice were used for all behavioral experiments (Figure 1). The moderate PAE model used here has been previously described (Brady et al., 2012, 2013). Briefly, following habituation, female C57BL/6J mice (Jackson Laboratory, Bar Harbor, ME) were gradually acclimated to drinking 10% (w/v) ethanol (EtOH) solution in a stepwise manner. Mice were first allowed to drink either 0.066% (w/v) saccharin or 5% (w/v) EtOH solution sweetened with 0.066% (w/v) saccharine for 4 h every day (from 10:00 to 14:00 h) for 4 days. Next, the concentration of EtOH solution was increased to 10% w/v sweetened with 0.066% (w/v) saccharin. This protocol has previously been shown to produce blood ethanol concentration (BEC) of 80–90 mg/dl (Brady et al., 2012, 2013) when tested at the end of the 4-h alcohol access period. After 7 days of drinking 10% EtOH or saccharin, female mice were placed in a cage along with a singly housed male for 2 h to enable mating immediately following drinking. The mating period lasted for 5 days during which female mice continued to have access to either 10% EtOH solution or saccharin control solution. Dams were weighed every 3–4 days to monitor weight gain, which indicates pregnancy. Following parturition, the access to EtOH solution or saccharin was weaned off using a step-down procedure over a 6-day period. Offspring produced from saccharin (SAC) and PAE dams were weaned at 21–23 days of age. The pups were then housed in same-sex pairs in cages in a temperature- and humidity-controlled environment under a reverse 12 h light/dark cycle (lights off at 8:00 h, on at 20:00 h). All the behavioral experiments were conducted during the dark phase in both male and female PAE and SAC control offspring (no more than 2/litter, *n* = 8 to 9 per sex/treatment; ~8–9 weeks at the onset of pretraining). All experimental procedures were performed in accordance with the National Institutes of Health Guide for Care and Use of Laboratory Animals and were approved by the University of New Mexico Health Sciences Center Institutional Animal Care and Use Committee.

### Operant apparatus

Behavioral flexibility was tested using a touchscreen-based discrimination and reversal learning paradigm, as described previously (Marquardt et al., 2014, 2020). Briefly, a sound- and light-attenuating box housed the operant chamber measuring 21.6 cm × 17.8 cm × 12.7 cm (model # ENV-307 W; Med Associates, St. Albans, VT). The floor of the chamber was a solid acrylic plate resting on the standard grid floor to facilitate ambulation. All chambers were equipped with both pellet dispensers (#F05684; Bio-Serv, Frenchtown, NJ) and peristaltic pumps (Lafayette Instruments, Lafayette, IN) to enable solid or liquid reward delivery into the magazine located at one end of the chamber. Beside the magazine is an ultra-sensitive lever. This end of the chamber is also equipped with a house light and a magazine light within the magazine. At the opposite end of the chamber is a touch-sensitive screen (Conclusive Solutions, Sawbridgeworth, United Kingdom) covered by a black acrylic aperture plate which allows two areas sensitive to touch (7.5 cm × 7.5 cm) which are separated by 1 cm and located at a height of 0.8 cm from the floor of the chamber. Stimulus presentation in the response windows and touches was controlled and recorded by the Limbic Software package (Conclusive Solutions).

### Pretraining

Male and female mice were gradually weight-reduced over a 10–14 day period and then maintained at 85% of their free-feeding body weight. Mice were then acclimated to the behavioral testing room and to the chosen reward over a 3–4-day period. Male and female PAE and SAC mice were randomly assigned to either the liquid or pellet reward groups. During pretraining and testing, mice received either precision food pellet reward (PR: 14 mg dustless pellets, Bio-Serv) or sweetened strawberry milk (LR: 30 μl, Nesquik. S.A., Vevey, Switzerland, non-fat milk). The reward was placed in a weigh boat in the home cage, and the total amount consumed was recorded daily. Mice were then habituated to the operant chamber to retrieve the reward out of the magazine in 30 min. Mice retrieving 10 rewards within 30 min were then moved to pretraining. First, mice were trained to obtain a reward by pressing a lever within the chamber. Mice pressing the lever and collecting 30 rewards in under 30 min were moved to the next stage, touch training, where the lever press led to the presentation of a stimulus (variously shaped and equi-luminescent) in either one of the response windows (spatially pseudorandomized). The touches in the window with no image had no response and the stimulus remained on the screen until nose poke on the window with the image elicited a response. Mice initiating, touching, and retrieving 30 rewards within 30 minutes were moved to the final stage of pretraining: punished training. This stage is similar to the touch training except the responses at a blank window during stimulus presentation were now considered errors and resulted in turning on the house light for a 15 s time-out period to discourage indiscriminate screen responding. While the correct responses resulted in moving on to the next trial, errors were followed by correction trials where the same stimulus was presented in the same left/right position until a correct response was made. Mice getting ≥75% correct responses (excluding correction trials) in under 30 min were moved on to the testing stage, the discrimination stage.

### Reward-based discrimination and reversal testing

All mice were tested on a pairwise discrimination-reversal paradigm where the mice learned to discriminate between two equi-luminescent stimuli followed by the reversal learning stage. The sessions lasted for a period of 60 min or until 30-first presentation trials were completed based on which criterion was reached first. During discrimination learning, two novel approximately equi-luminescent (Fan, Marbles) appeared on the touchscreen in a spatially pseudorandomized manner over 30-first presentation trials with a 5 s intertrial interval. The stimuli stayed on the screen until a response was made. Responses at one stimulus resulted in the reward (signaled by 1 s tone and magazine light turning on), while responses at the other stimulus resulted in a punishment time-out of 15 s (signaled by the house light turning on). Both the assignment of the initial stimulus and the reward type were randomized across treatment and both sexes. While the correct responses resulted in the reward followed by moving on to the next first presentation trial, errors on the first presentation trials were followed by correction trials where the same stimulus appeared on the same window until a correct response was made. Once the mice learned the stimulus reward association, where they were performing at ≥85% correct (excluding correction trials) over two consecutive days, they were considered to have reached the criterion and were moved onto the reversal stage. During the reversal stage, in order to test the ability to behave flexibly, the stimulus-reward contingencies were simply switched. Previously correct choice resulting in the reward is now the incorrect choice resulting in punishment time-out and vice versa. Mice were tested on 30-trial 1-h daily sessions similar to the discrimination stage. They are considered to have reached the criterion in this stage, once they have re-learned the new stimulus reward association and performed at ≥85% correct over 2 consecutive days (excluding the correction trials). The testing period ends once they reach the reversal criterion.

### Statistical analysis

For pretraining stages, the number of sessions required to pass each stage was analyzed by treatment (SAC vs. PAE) and reward (PR vs. LR) group. The following dependent measures were taken during discrimination and reversal: correct responses made, errors (i.e., incorrect responses made), and correction errors (i.e., correction trials made) which are a putative measure of perseveration during reversal (Brigman et al., 2013), stimulus-response (i.e., time from trial initiation to touchscreen response), and reward response (i.e., time from touchscreen response to reward retrieval). As correct and incorrect response measures were consistent in all analyses, incorrect responses and correction trials are reported throughout. Discrimination performance was analyzed across all sessions required to reach the criterion. In order to examine distinct phases of reversal (early perseverative and late learning), we separately analyzed errors and correction errors for sessions where performance was below 50% and performance from 50% to criterion, as previously described (Brigman et al., 2010b, 2013). The main effects of sex, treatment (PAE vs. SAC), reward (liquid vs. pellet), and interaction were compared for all measures using analysis of variance (ANOVA) followed by Tukey’s *post-hoc* test using Prism (GraphPad Prism 9.4.1, San Diego, CA).

## Results

### PAE intake

We found that the limited access paradigm yielded ethanol consumption levels in dams similar to those producing BACs of approximately 80–90 mg/dl (Brady et al., 2012, 2013). Offspring tested were taken from litters born to dams with an average consumption of 4.66 ± 0.09 g of EtOH/kg of body weight/day and 1% SAC. No significant differences were seen in alcohol consumption for PAE mice tested on PR vs. LR or by sex (Table 1). However, the total saccharin intake was significantly higher for SAC animals regardless of sex or reward (F1,64 = 69.19, *p* < 0.001; Table 1).

**Table 1:** Performance on training sessions (average sessions to criterion ± SEM).

Training stages	Liquid reward				Pellet reward			
	SAC		PAE		SAC		PAE	
	Male	Female	Male	Female	Male	Female	Male	Female
Lever press	2.10 ± 0.3	3.25 ± 1.0	2.27 ± 0.1	3.14 ± 0.8	3.25 ± 0.4	4.90 ± 0.9	2.75 ± 0.3	6.5 ± 0.7
Touch	4.3 ± 0.8	3.13 ± 0.5	3.18 ± 0.5	3.14 ± 0.3	3.25 ± 0.4	3.20 ± 0.5	2.63 ± 0.6	4.10 ± 0.4
punish	1.00 ± 0.0	1.00 ± 0.0	1.00 ± 0.0	1.00 ± 0.0	1.00 ± 0.0	1.10 ± 0.1	1.00 ± 0.0	1.20 ± 0.2

### Operant training

An analysis of the three-stage pretraining revealed a significant main effect of both sex (F1,64 = 16.17, *p* < 0.001) and reinforcer (F1,64 = 12.92, *p* < 0.001) during lever-press training. A *post-hoc* analysis showed male LR mice taking significantly fewer sessions than female PR mice. Following bar-press training, there were no significant differences by sex, reinforcer or treatment, or interactions in the touch training or punishment training stages (Table 1).

### Discrimination learning

**Figure 2:**
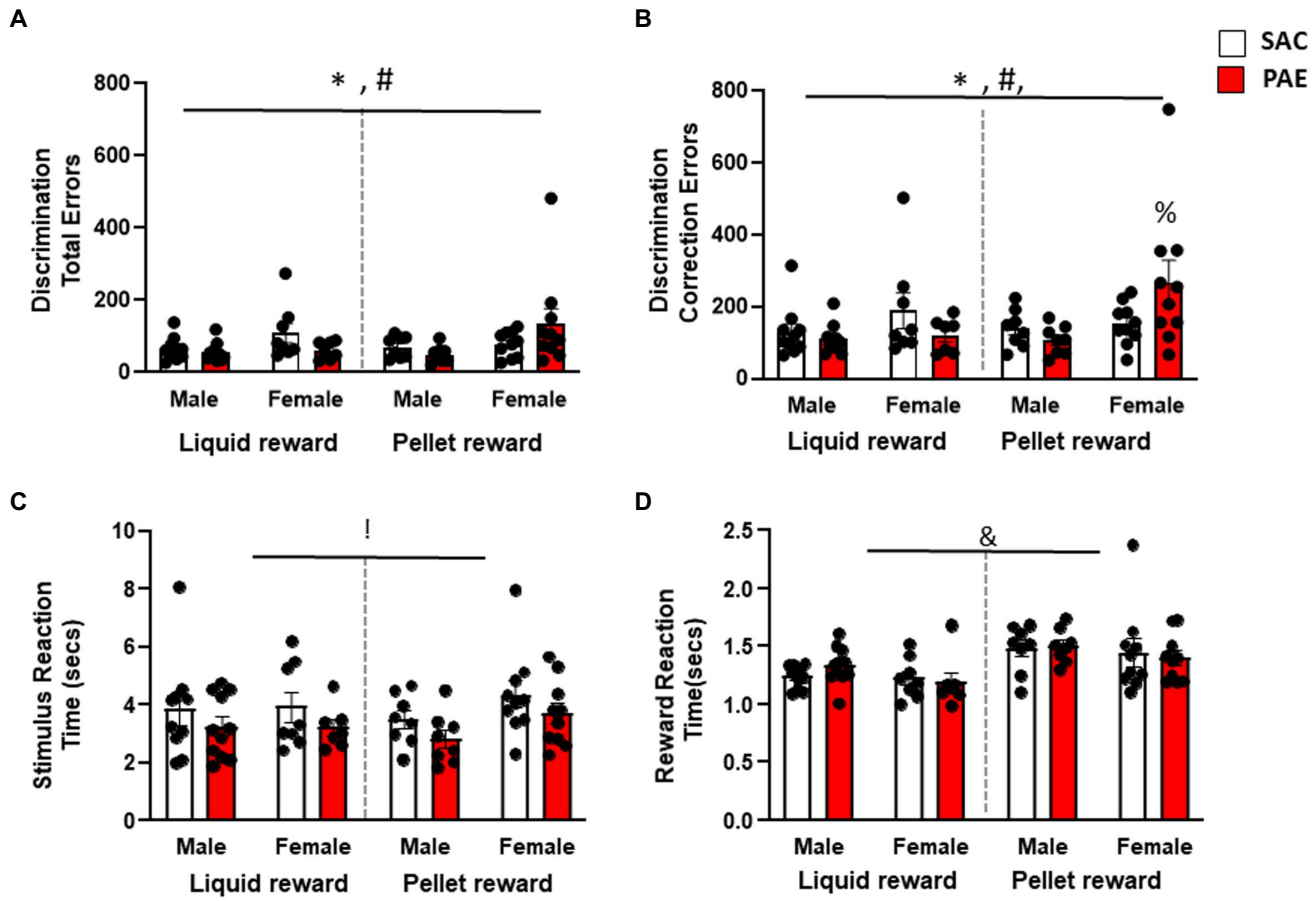
Sugar-sweetened liquid reinforcer (LR) is significantly more motivating than unsweetened pellet reinforcer (PR) during the initial phase of discrimination learning. **(A)** Female mice performed significantly more errors than male mice during the initial discrimination phase with female PAE PR mice making the most errors. **(B)** Female mice made significantly increased perseverative errors than the male mice during initial discrimination learning again driven by PAE PR female mice. **(C)** PAE mice had significantly reduced stimulus reaction times regardless of sex or reinforcer. **(D)** Liquid-reinforced mice had significantly shorter reward retrieval latencies when compared to pellet-reinforced mice. * = *p* < 0.05 main effect of sex, ! = *p* < 0.05 main effect of treatment, & = *p* < 0.05 main effect of reinforcer, † = *p* < 0.05 sex × reinforcer interaction, ‡ = *p* < 0.05 sex × treatment reinforcer, ∑ = *p* < 0.05 reinforcer × treatment, # = *p* < 0.05 sex × treatment × reinforcer; % = significant Tukey’s *post-hoc* test. Data are group mean ± SEM.

Consistent with previous reports, an analysis of pairwise discrimination learning revealed that female mice made significantly more errors (F1,64 = 7.27, *p* < 0.001; Figure 2A) and correction errors (F1,64 = 7.17, *p* < 0.001; Figures 2A,B) to attain criterion versus male mice. In addition, there was a significant sex × reinforcer × treatment interaction for errors (F1,64 = 4.10, *p* = 0.04) and correction errors (F1,64 = 3.95, *p* = 0.05). Male PAE mice made fewer errors and correction errors regardless of reinforcer group. In contrast, female PR-PAE mice made significantly more errors and correction errors than SAC controls, while female LR-PAE mice made significantly fewer errors and correction errors than the control (Figures 2A,B). Tukey’s *post-hoc* tests additionally revealed that the female PR-PAE mice had significantly more correction errors than male LR-SAC (*p* = 0.0458), male LR-SAC (*p* = 0.0094), and male PR-PAE (*p* = 0.0186) mice. Interestingly, an analysis of secondary measures found a significant main effect of treatment on reaction time (F1,64 = 5.00, *p* = 0.03; Figure 2C) with PAE mice having faster response times with no main effect of sex, reinforcer, or interactions. In contrast, an analysis of latency to retrieve rewards revealed a significant main effect of the reinforcer (F1,64 = 17.81, *p* < 0.0001; Figure 2D) with LR mice retrieving reward faster with no main effect of sex or treatment and no significant interactions.

### Reversal learning

**Figure 3:**
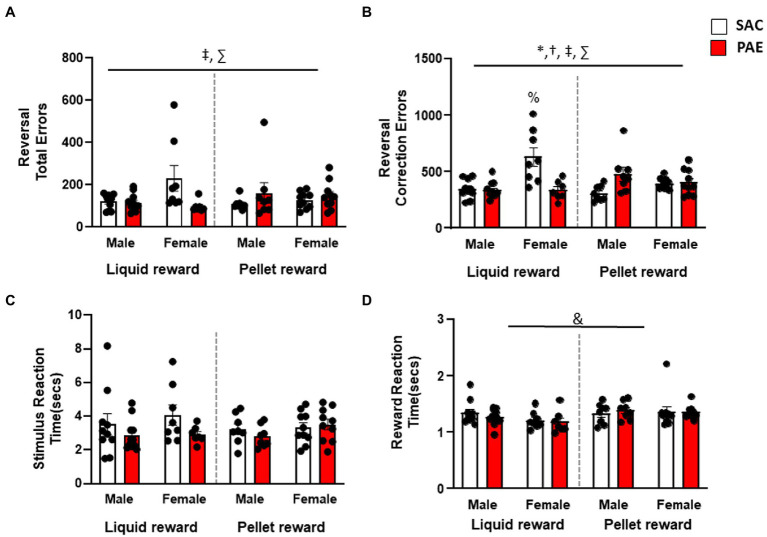
Reversal performance differed by sex and treatment depending on reward type. **(A)** Analysis of total errors during the reversal session showed that male PR-PAE mice were significantly worse than SAC controls, while female LR-SAC mice made significantly more errors than PAE female mice. **(B)** Analysis of total perseverative errors during the reversal session showed a similar pattern in which male PR-PAE mice were significantly worse than SAC controls, while female LR-SAC mice made significantly more errors than PAE female mice. **(C)** No significant differences were seen in stimulus reaction time across the reversal problem. **(D)** LR mice were significantly more motivated than the pellet-reinforced mice in both treatment groups and both sexes as measured by reward reaction time. * = *p* < 0.05 main effect of sex, ! = *p* < 0.05 main effect of treatment, & = *p* < 0.05 main effect of reinforcer, † = *p* < 0.05 sex × reinforcer interaction, ‡ = *p* < 0.05 sex × tretatment reinforcer, ∑ = *p* < 0.05 reinforcer × treatment, # = *p* < 0.05 sex × treatment × reinforcer; % = significant Tukey’s *post-hoc* test. Data are group mean ± SEM.

An analysis of errors made across the entire reversal problem revealed a significant reinforcer × treatment interaction (F1,64 = 7.28, *p* < 0.001) and a significant sex × treatment (F1,64 = 4.03, *p* = 0.04) effect for errors (Figure 3A). Both male and female PR-PAE mice performed more poorly than LR-SAC mice, with male LR-PAE mice performing significantly worse. In contrast, female LR-SAC mice performed significantly worse than female LR-PAE mice, while male LR mice did not significantly differ (Figure 3A). An analysis of correction trials found a significant main effect of sex (F1,64 = 6.64, *p* = 0.01) and a sex × reinforcer (F1,64 = 5.51, *p* = 0.02), sex × treatment (F1,64 = 15.56, *p* < 0.001), and reinforcer × treatment (F1,64 = 18.19, *p* < 0.001). Similar to total errors, male and female PR-PAE mice made more correction errors than LR-SAC mice, with male LR-PAE mice making significantly more perseverative errors. As in the error analysis, female LR-SAC mice made significantly more correction errors than femaleLR-PAE mice, while male LR mice did not significantly differ by treatment (Figure 3B). Furthermore, Tukey’s *post-hoc* test revealed that the female LR-SAC mice had significantly greater correction errors than all the other groups except male PR-PAE such as female LR-PAE (*p* = 0.0003), female PR-SAC (*p* = 0.0023), female PR-PAE (*p* = 0.0037), male LR-SAC (*p* = 0.0001), male LR-PAE (*p* < 0.0001), and male PR-SAC (p < 0.001) mice. An analysis of secondary measures found no significant difference in stimulus reaction time, while an analysis of latency to retrieve rewards again revealed a significant main effect of the reinforcer (F1,64 = 5.45, *p* < 0.002; Figures 3C,D) with LR mice retrieving rewards faster with no main effect of sex or treatment and no significant interactions (Figure 3D).

### Reversal stage analysis

**Figure 4:**
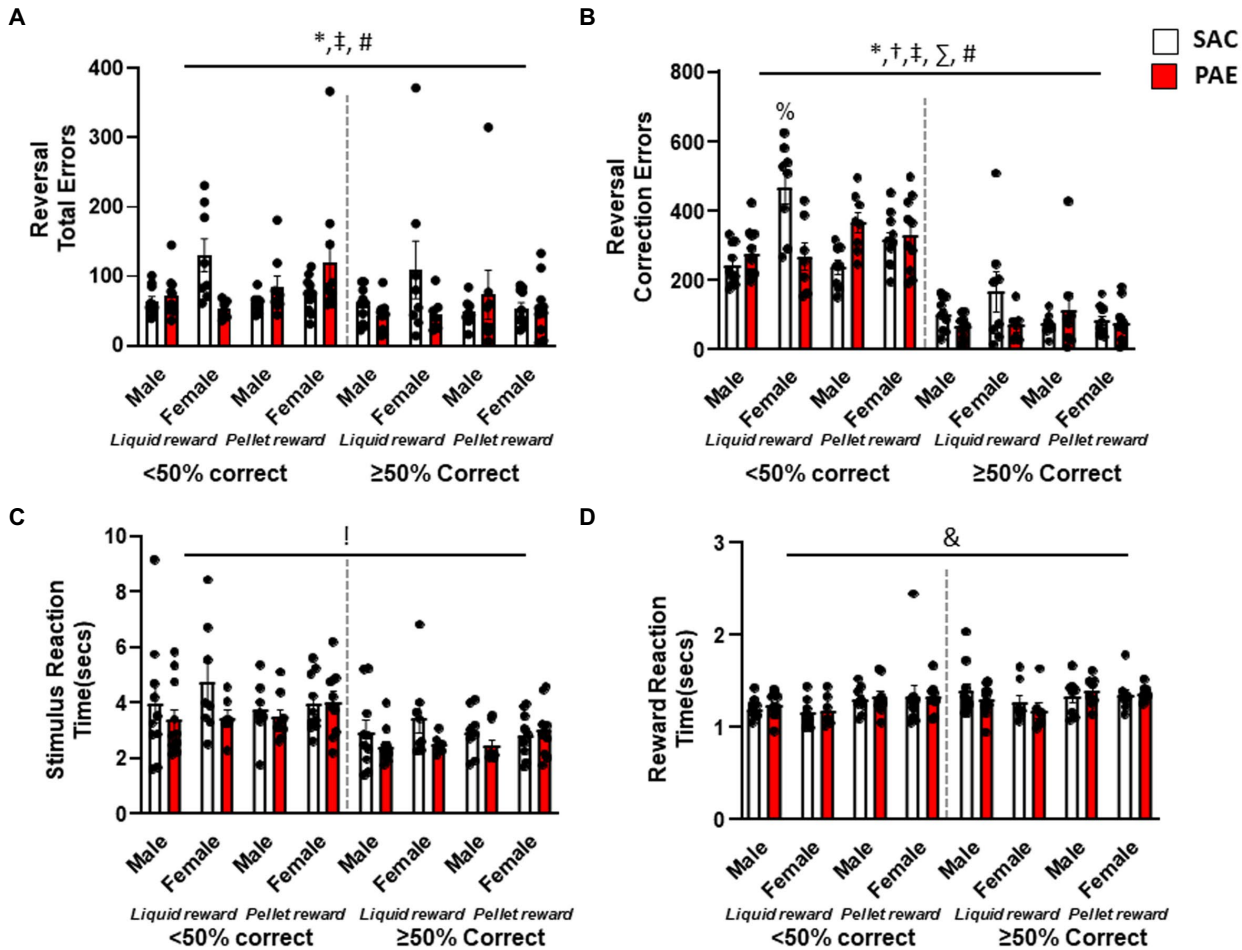
Sugar-sweetened liquid reinforcement changes the effects of moderate prenatal alcohol exposure. **(A)** Analysis of total errors during the early perseverative phase (when mice performed <50% correct) showed that female mice had significantly more errors when compared to male mice, with female LR-SAC mice making significantly more errors, while PR-PAE male and female? mice were significantly worse than the controls. **(B)** Similarly, male PR-PAE mice showed increased perseverative responding when compared to saccharine control mice but not on liquid reinforcement. No deficit following prenatal alcohol exposure was seen in female mice in either of the reinforcement groups, while female saccharine control mice on liquid reinforcer showed increased perseverative responding when compared to PAE mice. **(C)** No difference in motor behaviors was seen during the early perseverative phase, but PAE mice had faster stimulus reaction time when compared to saccharine control mice during the late reversal phase. **(D)** Liquid-reinforced mice were significantly faster to retrieve the reward during the early perseverative phase. * = *p* < 0.05 main effect of sex, ! = *p* < 0.05 main effect of treatment, & = *p* < 0.05 main effect of reinforcer, † = *p* < 0.05 sex × reinforcer interaction, ‡ = *p* < 0.05 sex × tretatment reinforcer, ∑ = *p* < 0.05 reinforcer × treatment, # = *p* < 0.05 sex × treatment × reinforcer; % = significant Tukey’s *post-hoc* test. Data are group mean ± SEM.

To examine the pattern of learning across the reversal problem, errors and correction errors were analyzed separately for early perseverative (<50% correct) and later learning (≥50%) sessions. An analysis of total errors during early perseverative sessions (Figure 4A) revealed a significant main effect of sex (F1,64 = 4.46, *p* = 0.0387), a reinforcer × treatment interaction (F1,64 = 9.35, *p* = 0.0033), and a sex × reinforcer × treatment interaction (F1,64 = 5.41, *p* = 0.0232; Figure 4A). Similarly, an analysis of correction errors during the early perseverative phase (Figure 4B) found a main effect of sex (F1,64 = 9.524, *p* = 0.003), a significant sex × reinforcer (F1,64 = 4.312, *p* = 0.04), sex × treatment (F1,64 = 16.53, *p* = 0.0001), and reinforcement × treatment (F1,64 = 13.18, *p* = 0.0006) effect for profound perseverative impairment in PAE mice. Furthermore, Tukey’s *post-hoc* test revealed that female LR-PAE mice had significantly more correction errors than all other groups except male PR-PAE such as female LR-PAE (*p* = 0.0013), female PR-SAC (*p* = 0.0127), female PR-PAE (*p* = 0.037), male LR-SAC (*p* < 0.0001), male LR-PAE (*p* = 0.004), and male PR-SAC (*p* < 0.0001) mice. An analysis of the later learning stage of reversal (Figure 4B) found no significant differences for total errors and a significant reinforcer × treatment effect for correction errors (F1,64 = 4.86, *p* = 0.031), whereby male LR-PAE mice made significantly more correction errors on learning, while female PR-SAC again made significantly more errors of this type. Reversal performance *via* correction errors once animals had attained chance found no main effect of sex, treatment, or reinforcer and no significant interactions (Figure 4B). Similar to discrimination performance, analysis of latency to retrieve reward revealed a significant main effect of reinforcer on sessions where performance was below 50% correct (F1,64 = 6.69, *p* = 0.04; Figure 4D) with no main effect of sex or treatment and no interactions. Interestingly, there was a significant main effect of treatment (F1,64 = 5.59, *p* = 0.04) on stimulus reaction time with PAE mice making significantly faster choices when performing above, but not below, 50% accuracy (Figure 4C).

### Trial-type analysis

**Figure 5:**
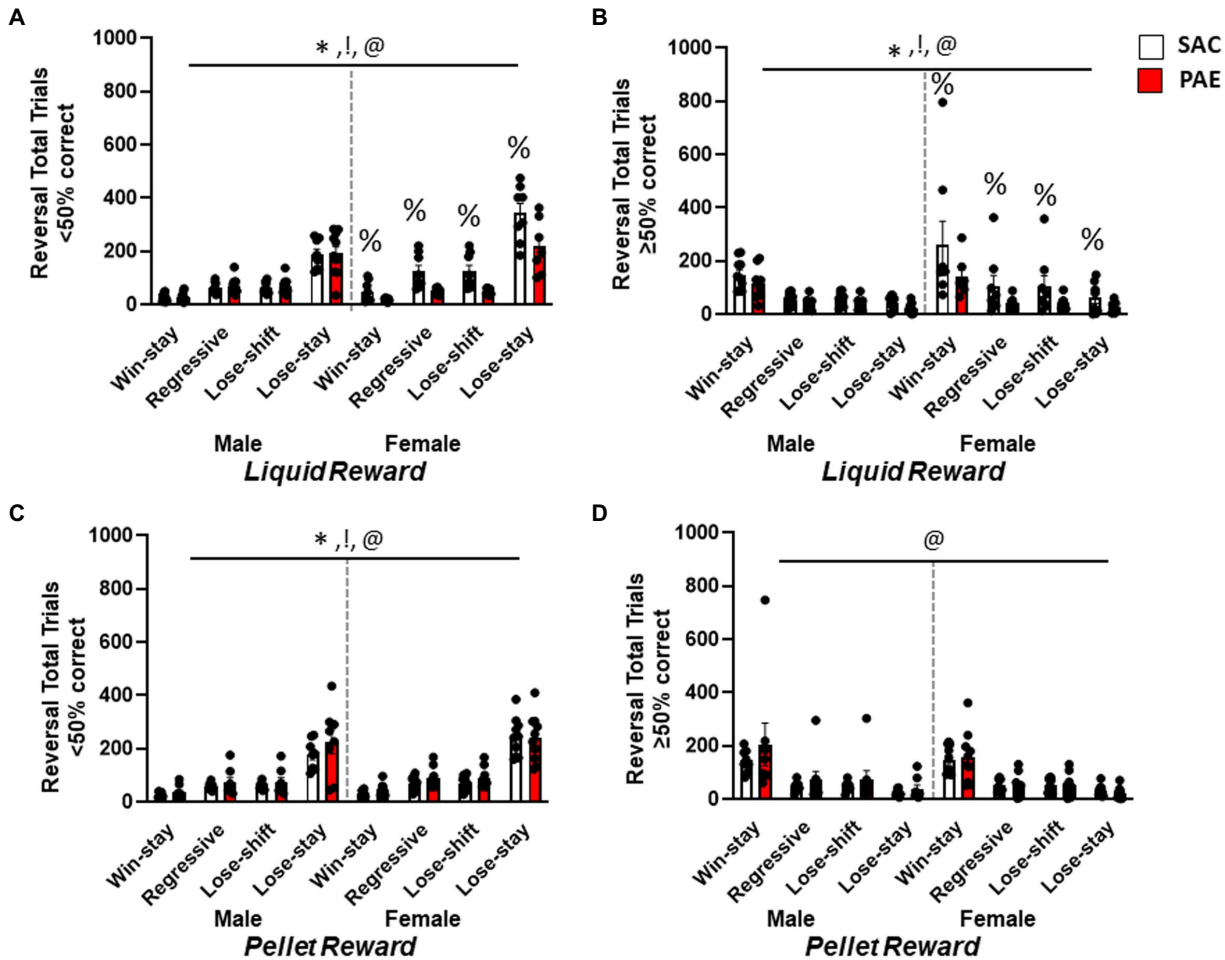
Female saccharine control mice had an increased number of all trial types during reversal learning. **(A)** Analysis of trial types for liquid-reinforced mice revealed a significant main effect of sex and treatment trial type whereby all mice made significantly more Lose→Stay trials than any other type. Female LR-SAC mice made a significantly increased number of Lose→Stay trial types when compared to liquid-reinforced female PAE mice. **(B)** In the liquid-reinforced group, during the learning phase (when mice were performing ≥50% correct), there was a significant main effect of sex, treatment, and trial type as the Win→Stay trial type predominated when compared to all other trial types. Female SAC LR mice had an increased number of total trials when compared to liquid-reinforced female PAE mice. **(C)** Analysis of trial types for pellet-reinforced mice showed a main effect of sex, treatment, and trial type whereby all mice made significantly more lose-stay trials, female mice made more of all trial types and PAE mice made significantly more of all trials. **(D)** When trial types were analyzed for learning sessions (when mice were performing ≥50% correct), there was a main effect of trial type whereby all mice made significantly more win-stay trials. * = *p* < 0.0005 main effects of sex, ! = *p* < 0.0005 main effect of treatment, @ = *p* < 0.0001 main effect of trial type; % = significant Tukey’s *post-hoc* test vs. SAC. Data are group mean ± SEM.

In order to characterize changes in behavioral patterns across the different trial types, data were analyzed as pairs of consecutive responses to determine the total number of correct responses followed by another correct response (win→stay), error responses followed by a correct response (lose→shift), correct responses followed by an error (regressive), and error responses followed by another error (lose→stay). The total number of each pair type was analyzed separately for reward type during early perseverative (<50% correct) sessions and later learning (≥50%) sessions. Analysis of the total number of trial types performed during the early perseverative phase in the LR group (Figure 5A) revealed the main effect of sex (F1,128 = 16.98, *p* < 0.0001), treatment (F1,128 = 16.86, *p* < 0.0001), trial types (F3,128 = 110.2, *p* < 0.0001), trial type × treatment (F3,128 = 4.35, *p* = 0.0059), and sex × treatment (F1,128 = 21.98, *p* < 0.0001) effects, whereby female mice performed more number of all trial types driven by female LR-SAC mice with more lose→stay responses (Figure 5A). An analysis during the later learning stage (Figure 5B) revealed the main effect of sex (F1,128 = 12.49, *p* = 0.006), treatment (F1,128 = 6.129, *p* = 0.0146), and trial types (F3,128 = 19.52, *p* < 0.0001). While lose→stay trials predominated during the early perseverative phase, as the mice re-learned the changed stimulus reward associations during the late learning stage, there was a change in the trial types being performed with an increase in win→stay trial type (Figures 5A,B). Female LR-SAC mice had an overall increase in the number of total trials being performed, specifically increased lose→stay trials during the early perseverative phase. A similar analysis performed in the PR group during the early perseverative phase (Figure 5C) revealed the main effect of sex (F1,128 = 4.014, *p* = 0.0449), treatment (F1,128 = 4.309, *p* = 0.0399), and trial types (F3,128 = 92.37, *p* < 0.0001) with increased lose→stay trial type predominating among both treatment groups in male and female mice, female mice performing more number of total trials when compared to male mice similar to the LR group. Male PR-PAE mice had a non-significant increase in the perseverative trial type, lose→stay when compared to the male PR-SAC mice (Figure 5C). Interestingly, in the PR group, PAE mice performed more trials when compared to the SAC mice but in the LR group with female LR-SAC mice performing an excessive number of all trial types, SAC mice had more total trial types than the PAE mice (Figures 4A,C). Furthermore, an analysis during the later learning stage (Figure 5D) showed the main effect of trial type (F3,128 = 24.20, *p* < 0.0001) with win→stay trial type predominating in both treatment groups in male and female mice.

### Correlational analysis with saccharine intake

**Figure 6:**
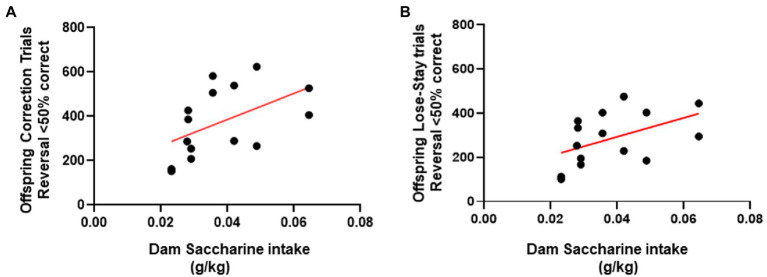
Female saccharine intake positively correlated with the perseverative trial types during the early perseverative reversal phase. **(A)** Saccharine intake in female mice positively correlated with the total number of correction errors performed during the early perseverative reversal phase. **(B)** Saccharine intake in female mice positively correlated with the total number of lose-stay errors performed during the early perseverative reversal phase. * = *p* < 0.05, *r*^2^ = 0.2621, # = *p* < 0.05, *r*^2^ = 0.2401.

To further investigate the unexpectedly increased perseverative behavior among the female LR-SAC mice, we performed the correlational analysis with the amount of maternal saccharine intake to the trial types. Female mice showed a positive correlation of saccharine intake to the number of correction errors (*r*^2^ = 0.2621, *p* = 0.0255; Figure 6A) and the total number of lose→stay trial types (*r*^2^ = 0.2401, *p* = 0.0319; Figure 6B), indicating that the maladaptive perseverative behavior might be related to the saccharine intake.

## Discussion

We have previously shown that moderate PAE during the first- and second-trimester equivalent is sufficient to impair reversal by increasing perseverative responding during the early stages of a pellet-rewarded reversal learning task (Marquardt et al., 2014, 2020). In the current study, we compared the efficacy of two reward types, high-sugar LR and low-calorie compressed food PR used previously. We found that liquid reinforcement drove faster initial training as LR mice were able to acquire the initial lever training at a significantly faster rate when compared to mice on PR in both sexes. We also show that LR mice were more reward focused, as measured by latency to retrieve rewards, through the entire course of the behavioral paradigm. Interestingly, male PAE mice rewarded with pellets (PR-PAE) again demonstrated impaired behavioral flexibility during early reversal learning (Marquardt et al., 2014, 2020). However, this effect was not seen in male LR-PAE mice. In contrast, female LR-SAC mice showed significantly *increased* perseveration during reversal. Together, our data underscore the importance of the motivational value of the reinforcers and suggest a critical need to examine how the reward content can alter performance and interact in unexpected ways with alcohol exposure and even saccharine during development.

Increasingly, the behavioral neuroscience community has focused on the standardization of behavioral paradigms to improve the reliability, reproducibility, and accuracy of translational neuroscience research (Steckler, 2015; Kim et al., 2017). The use of touchscreen-based operant testing with automated systems and computerized data collection has helped increase the standardization of testing protocols (Bussey et al., 2008; Brigman et al., 2010a; Horner et al., 2013; Dumont et al., 2021). However, the use of reinforcers, an essential motivational component of behavioral testing, has not been standardized (Kim et al., 2017; Phillips et al., 2017). Various types of liquid vs. solid reinforcers with different caloric values, fat and sugar content, and taste have been used in various studies. It has been repeatedly demonstrated that liquid rewards, particularly high-sugar strawberry milkshakes, are powerful reinforcers that enhance the performance of animals in operant touchscreen-based behavioral paradigms (Hutsell and Newland, 2013; Heath et al., 2015; Phillips et al., 2017). Although flavored pellets and sugar pellets have been used as reinforcers, to our knowledge, no study has directly compared their motivational value to highly potent liquid reinforcers. An added benefit of liquid rewards is that they avoid potential difficulties with solid food reinforcers such as dry mouth, difficulty in chewing, and satiety which can be particularly acute among inbred strains (Horner et al., 2013; Kim et al., 2017). Among liquid reinforcers, milk-based reinforcers such as high-sugar strawberry milkshakes are preferred over non-milk-based saccharine solutions (Phillips et al., 2017). In addition, liquid rewards with higher caloric content have been shown to speed operant training acquisition rates and reduce the duration of testing (Kim et al., 2017) which has led to them being widely adopted. As touchscreen-based approaches have become commonly used to test a host of behavioral domains, animal models of various disorders, and now cross-species translation approaches (Nithianantharajah et al., 2015; MacQueen et al., 2018; Cavanagh et al., 2021), the role that reinforcer types play in training and task performance becomes increasingly important.

Our current results show that mice on LR were significantly faster to pass the initial lever-press training with both PAE and SAC male and female LR mice having faster initial stage training versus PR mice. This observation is directly in line with several studies showing that liquid reinforcers, especially rewards with higher caloric value, had a faster operant training acquisition rate (Hutsell and Newland, 2013; Kim et al., 2017; Phillips et al., 2017). It is a commonly employed practice to reduce the rodents to 85% of their free-feeding weight to motivate them to perform the task (Marquardt et al., 2014) and internal states such as satiety and preference for the type of food reward can directly alter brain reward circuits (de Araujo et al., 2008). It is hypothesized that this difference in the level of motivation in turn affects the performance of the rodents on the operant tasks (Kim et al., 2017).

Consistent with our previous findings, our current data show that neither male nor female PAE mice differed from SAC mice on initial discrimination learning, although the analysis of secondary measures suggests reward type did alter motivation. LR mice had significantly shorter reward response times, suggesting that they were more motivated to perform the task when compared to PR mice. In addition, we also found sex-specific differences such as female mice taking longer to learn the initial discrimination association and making more errors than male mice, which is directly in line with our previous observation (Marquardt et al., 2014).

We have previously shown that both male and female PR-PAE mice exhibited behavioral inflexibility during the early reversal phase by performing an increased number of perseverative responses when compared to the saccharine control mice (Marquardt et al., 2014). Furthermore, single unit recording studies revealed that PAE decreased OFC while increasing DS firing rate during reversal learning. PAE also resulted in decreased oscillatory field activity between OFC and DS, thereby resulting in decreased coordinated activity between the two regions which is essential for efficient flexible behavior (Marquardt et al., 2020). Here, we found that male PR-PAE mice exhibited behavioral inflexibility as shown by an increase in correction trials during the early phase of reversal. However, male LR-PAE mice performed at levels comparable to controls, although both groups had similar average alcohol exposure *in utero*. Similar to discrimination performance, male LR mice had significantly shorter reward latencies compared to PR mice, suggesting a stronger motivation to retrieve reward during reversal. Together, the lack of perseverative responding and shortened reward latencies suggest that the highly motivational quality of the liquid reward was sufficient to overcome the effects of moderate PAE. Interestingly, enhancing motivational state has been shown to improve cognitive performance in rodents (Avlar et al., 2015) and humans (Nieto-Márquez et al., 2021) and has provided a framework for using intrinsic motivation states to improve cognitive abilities in neurodegenerative (Braver et al., 2014; Manera et al., 2017; Ruiz-González et al., 2021), neurodevelopmental (Prins et al., 2011; Demurie et al., 2012), and neuropsychiatric disorders (Choi and Medalia, 2010). The current data suggest that the motivational state may be a target for enhancing therapeutic efficacy in FASD.

Female PR-PAE mice showed a non-significant increase in errors and correction errors, while female LR-PAE mice showed no behavioral deficit during the early perseverative stage of reversal. Several factors may be contributing to the lack of significant impairment in female mice including the total exposure level in the current cohorts. Dams in the current study drank an average of 4.66 ± 0.09 g of EtOH/kg of body weight/day whereas our previous studies (Marquardt et al., 2014) utilized dams with an average drinking of 6.31 ± 0.34 g of EtOH/kg of body weight/day, suggesting that the critical exposure amount before deficits are seen may differ depending on the sex of the offspring. Perhaps most strikingly, we found that female LR-SAC mice showed the highest levels of perseveration, as measured by correction trials during the early phase of reversal. These levels were well outside what is typically seen in either male or female control animals when utilizing PR and were wholly unexpected because no study has reported any behavioral deficits in the control mice exposed to saccharine alone during the gestational period to our knowledge. We investigated if the level of saccharine intake by the dams could influence the performance of the pups and found that both male and female control mice had significantly increased amounts of saccharine intake when compared to the PAE mice (Table 2). This was because of the overall increase in fluid consumption by the control mice. The lack of bitter taste which was present in the saccharine-sweetened alcohol solution given to PAE mice and the improved taste of the drinking water by the addition of saccharine is thought to be the cause of increased fluid intake in the control mice and thus resulting in increased saccharine intake.

**Table 2:** Average intake (g/kg ± SEM).

Intake g/kg	Liquid reward				Pellet reward			
	SAC		PAE		SAC		PAE	
	Male	Female	Male	Female	Male	Female	Male	Female
Saccharine	0.047 ± 0.002	0.048 ± 0.004	0.027 ± 0.001	0.027 ± 0.001	0.052 ± 0.004	0.049 ± 0.003	0.027 ± 0.001	0.028 ± 0.001
EtOH	N/A	N/A	4.286 ± 0.164	4.481 ± 0.175	N/A	N/A	4.561 ± 0.169	4.624 ± 0.143

Saccharine intake in female LR-SAC mice showed a positive correlation with the number of perseverative errors and the lose-stay trial type, suggesting that the increase in maternal saccharine intake during pregnancy affected the cognitive ability of the pups, especially when engaging in goal-directed or stimulus-driven tasks rewarded with sweetened strawberry milk with high reward value. Pre-clinical studies examining the effects of prenatal exposure to sugar have shown that increased sugar consumption during pregnancy can alter the reward circuitry in the newborn. Specifically, dams given high fat, high-sugar diet 4 weeks prior and during the entire period of gestation had offspring that later exhibited an increased preference for palatable, high-caloric food driven by the permanent changes in the mesolimbic reward system such as increased expression of mu-opioid receptor mRNA and decreased expression of dopamine transporter DAT (Ong and Muhlhausler, 2011). Increased maternal sugar consumption has also been found to increase apoptosis and alter apoptotic signaling factors in the hippocampus of rats exposed to prenatal high-sugar diets concomitant with deficits in spatial acquisition tasks (Kuang et al., 2014). Data from human studies have shown similar findings. Project Viva, a prospective observational cohort study in humans conducted to understand the effects of both increased maternal and child sugar consumption on the child’s cognition, found that increased maternal sugar consumption particularly from sugar-sweetened beverages during pregnancy negatively impacted children’s learning and memory (Cohen et al., 2018). Together, these data suggest that prenatal exposure consumption of high sugar can negatively impact cognitive abilities. In contrast, little is known about how PAE alters hedonic value for rewards during adolescence and adulthood. While it has been shown that prenatal exposure in humans can lead to increased appetitive responses to alcohol-associated cues later in life, less is known about responses to non-alcohol rewards (Faas et al., 2015). In one rodent study, male mice exposed to alcohol *via* an *ad libitum* liquid diet during gestation and then given chronic unpredictable stress were shown to have significantly decreased intake of high-content sucrose during preference testing, but how PAE alone affects preference for sweetened rewards has not been examined in detail (Hellemans et al., 2010).

In our current study, dams were exposed to saccharine which is a non-nutritive sweetener (NNS), which has comparable hedonic value as sugar while being calorie deficient. Nutrient intake is regulated by both the hedonic value and nutrient value of the food, which are encoded by different neuronal circuitry and the dorsal and ventral striatum, respectively. NNS, having no nutritive value, may have differential effects on the satiety center and post-ingestion neuroendocrine signaling that could later influence preferences for sweetened rewards *via* alterations in glycemic control and altered gut microbiome. This is supported by a randomized cross over trial which found that sucralose, a NNS, altered both appetite and reward processing using fMRI (Yunker et al., 2021). Female participants with obesity on NNS consuming more calories and showing greater neuronal responses to food cue in the medial frontal cortex and orbito-frontal cortex. NNS consumption may also affect offspring during development and infancy, as studies have confirmed that NNS can be transferred to the fetus through the placenta and to the infant *via* breast milk (Palatnik et al., 2020). Prenatal and postnatal exposure to NNS has been shown to significantly alter the microbiome and metabolic alterations in rodents and in humans (Englund-Ögge et al., 2012; Olivier-Van Stichelen et al., 2019). Specifically, maternal consumption of NNS during pregnancy and lactation enhances sweet preference and lowers the preference thresholds (Zhang et al., 2011; Chen et al., 2013) and metabolic dysregulation in the offspring (Araújo et al., 2014; Olivier-Van Stichelen et al., 2019).

Overall, our findings of intact reversal learning for low-sugar food pellets and impaired reversal for high-sugar liquid reinforcers in female mice exposed to NNS during development suggest an important role for motivated cognition in reversal learning. Cognition and motivation are not separate subsystems but rather integrated processes (Yarrow and Messer, 1983; Hughes and Zaki, 2015; Madan, 2017) involved in goal-directed behavior across species (Chiew and Braver, 2011; Braver et al., 2014; Botvinick and Braver, 2015; Bergstrom et al., 2018). Corticostriatal networks involved in behavioral flexibility (Brigman et al., 2013; Marquardt et al., 2017), working memory (Gilbert and Fiez, 2004), inhibitory control (Gilbert and Fiez, 2004; Diamond, 2013), and temporal cognition (Avlar et al., 2015) have been shown to process information regarding motivational states. Human imaging studies have shown that the lateral prefrontal cortex acts as a confluence zone where the information regarding cognition and motivation is integrated (Braver et al., 2014; Botvinick and Braver, 2015), and motivational state is thought to drive cognitive processing in the prefrontal cortex by affecting the preparatory processing such as selective attention, perception, and action selection (Hughes and Zaki, 2015). Furthermore, the neuromodulatory effects of dopamine both at cellular and circuit levels in the cortex and striatum have been strongly implicated as a candidate for mediating the cognition–motivation interaction (Braver et al., 2014). To date, it is not well understood how differences in the motivational state might affect behavioral flexibility in rodents, but there is evidence to suggest that motivation for reward plays a large part in determining the performance in behavioral tasks such as reversal learning. Our findings, that SAC Female mice have discrimination rates similar to control regardless of reinforcer and significantly impaired reversal only to high-sugar rewards, suggest that gestational exposure to NSS that improves motivated learning may conversely impair flexible choices when motivation is high. In addition, the hedonic value of the rewards used may have profound effects on results with models of neurodevelopmental insults including PAE by altering brain reward circuitry (de Araujo et al., 2008).

In conclusion, we found that moderate PAE results in maladaptive perseveration, and this behavioral response is dependent on the motivating factors present such as the reinforcer type. Sugar-sweetened liquid reinforcers are more powerful and have better motivational value when compared to unsweetened solid reinforcers with lesser hedonic value. These results suggest that prenatal saccharine exposure alters the response to high-sugar rewards during adulthood and underlines the importance of understanding the influence of hedonic systems in driving the motivational state of rodents being trained on operant tasks. While there are no current standards set for NNS intake during pregnancy, our current results also underline the need to carefully examine the effects of NNS, and NNS plus alcohol intake on later behavior in offspring.

## Data availability statement

The raw data supporting the conclusions of this article will be made available by the authors, without undue reservation.

## Ethics statement

The animal study was reviewed and approved by University of New Mexico Health Sciences Center Institutional Animal Care and Use Committee.

## Author contributions

JC designed and conducted experiments, analyzed data, and wrote the manuscript. BJ and JW conducted experiments and analyzed data. JB conceived the research, analyzed data, and wrote the manuscript. All authors contributed to the article and approved the submitted version.

## Funding

This manuscript was supported by the National Institute on Alcohol Abuse and Alcoholism grants P50AA022534 and R01-AA025652. Data are available upon request from the authors.

## Conflict of interest

The authors declare that the research was conducted in the absence of any commercial or financial relationships that could be construed as a potential conflict of interest.

## Publisher’s note

All claims expressed in this article are solely those of the authors and do not necessarily represent those of their affiliated organizations, or those of the publisher, the editors and the reviewers. Any product that may be evaluated in this article, or claim that may be made by its manufacturer, is not guaranteed or endorsed by the publisher.
